# Negative feedback regulation of calcineurin-dependent Prz1 transcription factor by the CaMKK-CaMK1 axis in fission yeast

**DOI:** 10.1093/nar/gku684

**Published:** 2014-07-31

**Authors:** Eugenia Cisneros-Barroso, Tula Yance-Chávez, Ayako Kito, Reiko Sugiura, Alba Gómez-Hierro, David Giménez-Zaragoza, Rosa Aligue

**Affiliations:** 1 Departament de Biologia Cellular, Immunologia i Neurociències, Facultat de Medicina, Universitat de Barcelona, Institute of Biomedical Research August Pi i Sunyer (IDIBAPS), Barcelona 08036, Catalunya, Spain; 2 Laboratory of Molecular Pharmacogenomics, School of Pharmaceutical Sciences, Kinki University, Kowakae, Higashi-Osaka 577–8502, Japan

## Abstract

Calcium signals trigger the translocation of the Prz1 transcription factor from the cytoplasm to the nucleus. The process is regulated by the calcium-activated phosphatase calcineurin, which activates Prz1 thereby maintaining active transcription during calcium signalling. When calcium signalling ceases, Prz1 is inactivated by phosphorylation and exported to the cytoplasm. In budding yeast and mammalian cells, different kinases have been reported to counter calcineurin activity and regulate nuclear export. Here, we show that the Ca^2+^/calmodulin-dependent kinase Cmk1 is first phosphorylated and activated by the newly identified kinase CaMKK2 homologue, Ckk2, in response to Ca^2+^. Then, active Cmk1 binds, phosphorylates and inactivates Prz1 transcription activity whilst at the same time *cmk1* expression is enhanced by Prz1 in response to Ca^2+^. Furthermore, Cdc25 phosphatase is also phosphorylated by Cmk1, inducing cell cycle arrest in response to an increase in Ca^2+^. Moreover, *cmk1* deletion shows a high tolerance to chronic exposure to Ca^2+^, due to the lack of cell cycle inhibition and elevated Prz1 activity. This work reveals that Cmk1 kinase activated by the newly identified Ckk2 counteracts calcineurin function by negatively regulating Prz1 activity which in turn is involved in activating *cmk1* gene transcription. These results are the first insights into Cmk1 and Ckk2 function in *Schizosaccharomyces pombe.*

## INTRODUCTION

Calcium is a secondary messenger that is involved in the regulation of nearly every aspect of cellular life. Calcium signalling is first decoded by the EF-hand family of proteins, which is characterised by the presence of a helix-loop-helix Ca^2+^ binding domain. Calmodulin (CaM) is a small ubiquitous adaptor protein that belongs to the EF-hand family. In CaM, conformational change is driven by Ca^2+^-binding to its four EF-hands, and thereby triggering the capacity of CaM to interact with different proteins, including the Ca^2+^/CaM-dependent kinase (CaMKK) family of proteins and phosphatase calcineurin (1).

The Ca^2+^/CaM-dependent protein kinase family is involved in many cellular responses that are triggered by elevated intracellular Ca^2+^ concentrations (2). The family includes CaMKI, a monomeric kinase that is activated by Ca^2+^/CaM, and also by phosphorylation. Like most other Ser/Thr protein kinases, CaMKI has an ‘activation loop’ phosphorylation site (T174 to T180, depending on the mammalian isoforms) (3,4). When Ca^2+^/CaM binds to CaMKI the activation loop site is exposed, enabling phosphorylation by the upstream Ca^2+^/CaMKK when it is simultaneously activated by Ca^2+^/CaM (4,5). Thus, CaMKK/CaMK signalling cascades are regulated at multiple levels by Ca^2+^/CaM (6–8).

In *Schizosaccharomyces pombe*, Cmk1 has been identified as a member of the CaM-dependent protein kinase cascade (9). The *cmk1 gene* encodes a 335-amino acid protein significantly homologous to mammalian CaMK1, including a putative phosphorylation site (Thr-192) for CaMKK. Cmk1 activity is dependent on Ca^2+^ and CaM, and increases when threonine 192 is mutated to aspartic acid (Cmk1-T192D), which is consistent with the conclusion that Cmk1 activity is regulated in a manner homologous to mammalian CaMK1 (9–11). It has also been reported that the overexpression of Cmk1-T192D causes cell cycle arrest (9). However, the molecular mechanics of Cmk1 activity remains unknown.

The kinase Ssp1 has been postulated as the fission yeast CaMKK orthologue. As in animal cells, Ssp1 phosphorylates and activates the catalytic subunit of AMPK (Ssp2) in its activation loop (Thr-189) when cells are starved of nitrogen or glucose (12,13). However, the Ca^2+^/CaM-dependent activity of Ssp1 or Cmk1 regulation by Ssp1 have not yet been described.

The Ca^2+^/CaM-dependent phosphatase calcineurin, Ppb1 in *S. pombe*, is known to be one of the central elements of calcium response in a wide variety of cell types and organisms. Like CaM-dependent kinases, calcineurin is involved in various Ca^2+^-mediated processes in human cells, including lymphocyte activation, cardiac hypertrophy, apoptosis, angiogenesis and memory development (14–16). In many cellular events, calcineurin functions by dephosphorylating a zinc finger transcription factor, Crz1 in budding yeast and Prz1 in fission yeast (17). In an analogous manner, calcineurin regulates the mammalian NFAT transcription factors which contain a Rel-homology region (RHR) to bind to DNA.

After CaM binds to Ca^2+^, it binds to Ppb1, enabling dephosphorylation, activation and nuclei shuttling of the Prz1 transcription factor. Once Prz1 is in the nuclei, it regulates the expression of genes involved in the control of Ca^2+^ homeostasis (18,19). When an increase in Ca^2+^ is prevented or calcineurin is inhibited in budding yeast and human cells, Crz1 and NFAT, respectively, are rephosphorylated by kinases, such as: casein kinase I (CK1, Hrr25) (20,21); the cyclic AMP-dependent protein kinase A (PKA) (22–24); the CDK, Pho85 (25); glycogen synthase kinase 3 (GSK3) (26); dual specificity tyrosine-phosphorylation-regulated kinase (DYRK) (27); MAPK p38 and JNK (28,29). Crz1 and NFAT are then rapidly exported to the nucleus and calcineurin-dependent gene expression is terminated. Thus, many protein kinases interact with calcineurin-dependent transcription factors to inactivate transcriptional activity. Most of these kinases have been studied in human T-cells, *Drosophila* and budding yeast.

In fission yeast, a genetic screen to isolate negative regulators of Prz1 transcription factor identified seven genes (30), two negative regulators, the 14-3-3 proteins Rad24 and Rad25, and five putative regulators, Pka1, Msn5, Pac1, Tfs1 and Ape2 (30). Rad24 and Rad25 bind to phosphorylation sites of Prz1 and inhibit the dephosphorylation of Prz1 by calcineurin. Pka1 represses the CDRE transcriptional activity of Prz1, although not by direct phosphorylation. Msn5 regulates Prz1-mediated transactivation by promoting its nuclear export (30). Nothing has been reported to date regarding the mechanism underlying the negative regulation of Prz1 by Pac1, Tfs1 and Ape2.

Here, we focus on the physiological function of Cmk1 in response to an increase in cellular Ca^2+^. We found that Cmk1 is a target of the Prz1 transcription factor, which in turn is involved in negatively regulating Prz1 activation in Ca^2+^ signalling. In addition, we have identified a second CaMKK in fission yeast, the Ckk2 kinase, which is involved in the activation of Cmk1 in response to Ca^2+^.

## MATERIALS AND METHODS

### Fission Yeast Strains, Media and Techniques

Strains were manipulated using standard techniques (31,32) and are listed in Supplementary p S1. Gene deletion and epitope tagging were carried out as described elsewhere (33). For Ca^2+^ sensitivity assays, 10-fold serial dilutions of exponentially growing cells were spotted onto agar plates and treated with the indicated dose of CaCl_2_.

### Plasmid Construction

The *cmk1* coding sequence was polymerase chain reaction (PCR)-amplified from *S. pombe* cDNA using specific primers and cloned into the XhoI/ BamHI site of the *nmt* (no message in thiamine)-driven expression vector pREP3x to create the pREP3x-cmk1. Then, the PstI/NotI fragment of the pREP3x was ligated to the pREP1 vector to obtain pREP1-cmk1. To analyse the effect of the different mutants, single point mutations were introduced using pREP1-cmk1 as a template and the oligonucleotides listed in Supplementary Table S2.

### Western Blot Analysis

Whole-cell extracts were obtained from exponentially growing cells by mechanical disruption in standard NP-40 lysis buffer (50 mM Tris (pH 8.0), 150 mM NaCl, 2.5 mM EDTA, 0.002% NP-40, 50 mM NaF, protease inhibitor tablet (Complete Mini EDTA-free tablets, Roche, Indianapolis, IN)). The GFP-Prz1 protein was immunoprecipitated from cell extracts with 1 μg of monoclonal anti-GFP antibody (Abcam, Cambridge, UK) and using 30 μl of protein A/G Sepharose beads (Pierce, Rockford, IL). Protein was resolved by SDS-PAGE using 10% gels with an acrylamide/bisacrylamide ratio of 99:1. Proteins were transferred to nitrocellulose membranes and the membranes were blocked with 5% milk in Tris-buffered saline with 0.05% Tween. The antibody probes used for immunoblotting were: monoclonal anti-HA (12CA5, Roche, Indianapolis, IN; 1/1000); polyclonal anti-GFP (Abcam, Cambridge, UK; 1/5000); polyclonal anti-PSTAIR (Upstate Biotechnology, Lake Placid, NY; 1/1000), polyclonal anti-phospho-Akt, which recognises phosphorylated consensus R-XX-S/T (1/1000, Cell Signaling Technology, Beverly, MA) and polyclonal anti-cdc25 (1/1000). Horseradish peroxidase–conjugated anti-mouse or anti-rabbit antibodies (Bio-Rad, Richmond, CA) were used as secondary antibodies. Membranes were developed by enhanced chemiluminescence (ECL kit, Amersham-Pharmacia, Piscataway, NJ). The λ-phosphatase (NEB) was used for phosphatase treatment. Cell extracts were incubated at 30ºC for 1 h in the presence or absence of λ-phosphatase. As a control, λ-phosphatase activity was inhibited with 5 mM Na_2_VO_4_ and 50 mM NaF. Cells were incubated when required, with the calcineurin-inhibitor FK506 (Sigma F4679) (0.5 μg/ml) for 1 h.

### Quantitative real-time PCR

Total RNA was isolated using RNeasy plus mini kit according to the manufacturer's instructions (Qiagen, Crawley, UK). RNA quality and concentration was determined using a Nanodrop 1000 Spectrophotometer (Thermo Fisher Scientific, Waltham, MA). cDNA was synthesised with the High-Capacity cDNA Reverse transcription kit (Applied Biosystems, Foster City, CA) using random primers. Relative quantification of cDNA was carried out in duplicate on an Mx3000P qPCR (Stratagene, La Jolla, CA) using Express SYBR® GreenER^TM^ qPCR Supermix Universal (Invitrogen, Carlsbad, CA). qPCRs were denatured at 95°C for 10 min, followed by 40 cycles of 95°C for 30 s, 60°C for 30 s, and 72°C for 1 min. The oligonucleotides used for qPCR amplification are listed in Supplementary Table S2. The *act1* gene encoding actin was used to normalise. For purposes of plotting graph, data were also normalised to wild-type without treatment values. The data shown are representative of three independent experiments.

### Calcineurin-dependent Response Element (CDRE)-dependent Reporter Assay

The cells were transformed or integrated with the CDRE reporter plasmid (34) and were untreated or treated with CaCl2. For the experiments with overexpression of Cmk1, wild-type cells carrying CDRE-reporter (34) were transformed either with pREP1-Cmk1 or empty pREP1 and were incubated in EMM without tiamine to activate the *nmt* promoter. The cells were incubated with 0.5 mM D-luciferin at 30°C and CDRE transcriptional activity was measured as described previously (34).

### Fluorescence microscopy

For GFP visualisation, cells were grown to their exponential phase. A Zeiss Axiovert 200M inverted microscope with a 100X oil-immersion objective was used to visualise the cells. Images were taken with a Coolsnap HQ camera and were processed using Slidebook TM 3.0.1 software.

## RESULTS

### Cmk1 is regulated both at the transcriptional and post-translational level by Ca^2+^.

**Figure 1.**
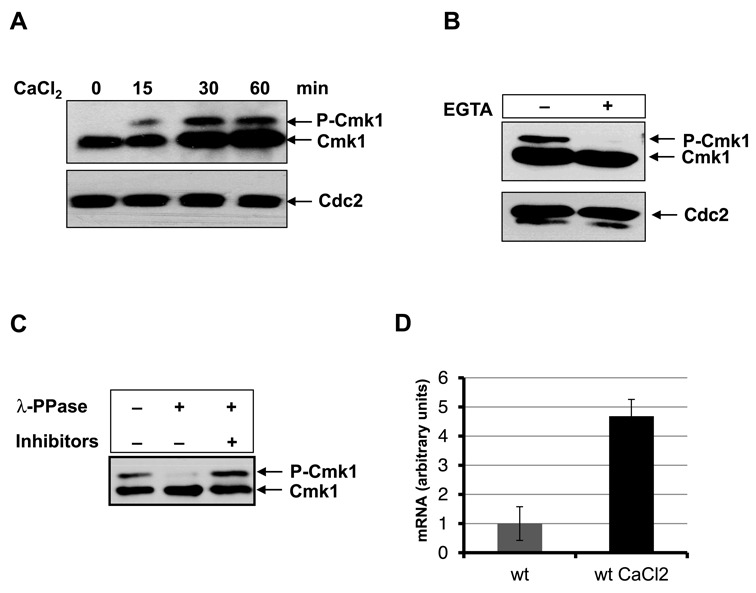
Increase of Cmk1 expression and phosphorylation due to Ca^2+^. (**A**) Time course of cells harbouring endogenous *cmk1:HA* gene exposed to 100 mM CaCl_2_. Cmk1 protein level was analysed at the times indicated by Western blot using anti-HA antibodies (top) and Cdc2 was probed as a loading control with anti-PSTAIR antibodies (A and B, bottom). (**B**) *cmk1:HA* cells exposed to 100 mM CaCl_2_ for 30 min were treated (+) or untreated (-) with EGTA before analysing the Cmk1 protein by Western blot using anti-HA antibodies (top). (**C**) Cell extracts of cells expressing Cmk1-HA in the presence of 100 mM CaCl_2_ were treated before electrophoresis with λ-protein phosphatase and with phosphatase inhibitors when indicated. Cmk1 was analysed by Western blot using anti-HA antibodies. (**D**) Cmk1 gene expression. The mRNA of cells treated (wt CaCl_2_) or untreated (wt) with 100 mM CaCl_2_ was extracted and the *cmk1* mRNA level was analysed by RT-PCR using the oligonucleotides Cmk1 RT Fw and Rv.

The Cmk1 protein was analysed in response to Ca^2+^. We found that Cmk1 protein levels increased during exposure to Ca^2+^ (Figure 1A). In addition, we observed the appearance of a slow migration band concomitant with exposure to Ca^2+^ (Figure 1A). To confirm the Ca^2+^-dependence of the slow Cmk1 migration band, growing cells were treated with Ca^2+^ and divided into two cultures, with Ca^2+^-chelator EGTA added to one of them. As Figure 1B shows, the slow migrating band was eliminated when EGTA was added to Ca^2+^-treated cells. To determine whether phosphorylation contributes to the Cmk1 slow migration band, we performed a phosphatase assay. Cmk1-HA was immunoprecipitated from Ca^2+^-treated extracts and incubated with or without λ-protein phosphatase. The Cmk1 mobility shift was then analysed. As shown in Figure 1C, phosphatase treatment greatly reduced the presence of the slow migration band, indicating that the Cmk1 protein is phosphorylated in response to Ca^2+^.

Ca^2+^-induced transcription is the principal process that has been characterised in *S. pombe*. We studied whether the expression of Cmk1 induced by Ca^2+^ is controlled at the transcriptional level. To test this possibility, we monitored *cmk1* mRNA, detecting an increase in *cmk1* mRNA levels following Ca^2+^ treatment (Figure 1D). Together, these results suggest that Ca^2+^ regulates Cmk1 expression and phosphorylation.

### Phosphorylation of Cmk1 in response to osmotic stress is due to Ca^2+^ signalling.

**Figure 2.**
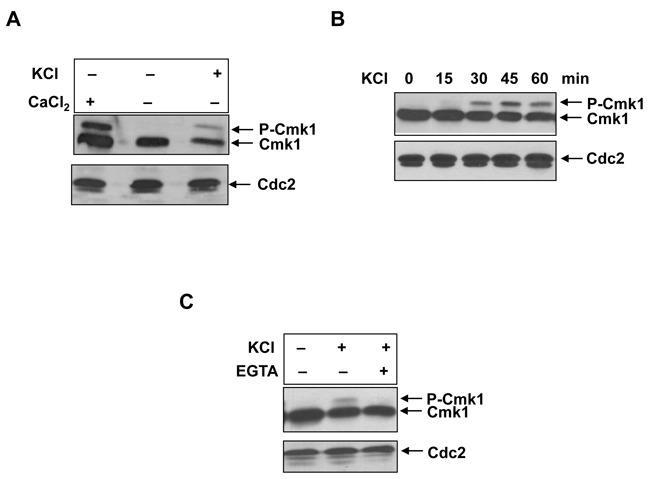
Phosphorylation of Cmk1 in response to osmotic stress. (**A**) *cmk1:HA* strain was treated with 100 mM CaCl_2_ or 1M KCl when indicated and Cmk1 phosphorylation was detected by Western blot with anti-HA antibodies (top). (**B**) Time course of cells harbouring endogenous *cmk1:HA* gene exposed to 1M KCl. Cmk1 protein level was analysed at times indicated as in A (top). (**C**) The same strain *cmk1:HA* was treated with 1M KCl and 20 mM EGTA when indicated and Cmk1 was detected as in A (top). Anti-PSTAIR antibodies were used to probe Cdc2 as a loading control (bottom).

An increase in extracellular Ca^2+^ can also provoke osmotic stress in cells; therefore, we also analysed the Cmk1 protein in response to osmotic stress by KCl. The Cmk1 protein was also phosphorylated in the presence of KCl, displaying a slow migration band as in the response to Ca^2+^ (Figure 2A). Cmk1 phosphorylation was concomitant with KCl exposure, as shown in the time course of Figure 2B.

It has been reported that high extracellular concentrations of KCl and NaCl cause an increase in intracellular Ca^2+^, as a result of the stimulation of extracellular Ca^2+^ intake (34). In order to determine whether Cmk1 phosphorylation in the presence of KCl is due to osmotic stress independently of or dependently on an increase in cytosolic Ca^2+^, we treated the cells with KCl with or without EGTA. The slow migration band did not appear when EGTA was added to the medium, confirming that phosphorylation of Cmk1 in response to KCl is a consequence of an increase in the intracellular Ca^2+^ concentration rather than of osmotic stress *per se* (Figure 2C).

### Cmk1 gene expression is regulated by the calcineurin-dependent Prz1 transcription factor

**Figure 3.**
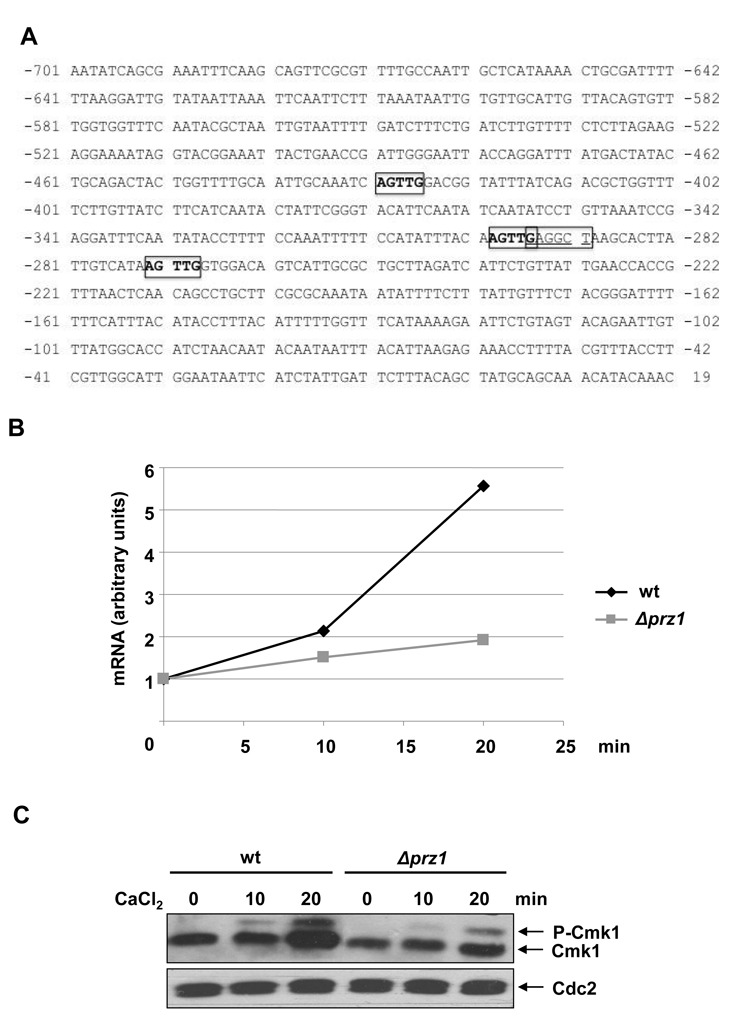
Transcription of Cmk1 is regulated by Prz1 in response to Ca^2+^. (**A**) Promoter analysis of the *cmk1* gene. Calcineurin-dependent response element (CDRE), AGCCTC/GAGGCT is boxed and putative Ca^2+^-dependent response elements CAACT/AGTTG are boxed in bold. (**B**) Cmk1 gene expression was analysed in wild-type (wt) and *prz1* deleted *(Δprz1*) cells treated with 100 mM CaCl_2_ at different times. The mRNA of the cells was extracted and the *cmk1* mRNA level was analysed by RT-PCR using the oligonucleotides Cmk1 RT Fw and Rv. (**C**) Cmk1 protein level was analysed from the same cells as in B by Western blot with anti-HA antibodies (top). Anti-PSTAIR antibodies were used to probe Cdc2 as a loading control (bottom).

An examination of the *cmk1* promoter revealed the presence of a number of putative Ca^2+^-dependent response elements: CAACT/AGTTG located at 431–427, 300–296 and 273–269 bp upstream of ATG (Figure 3A), suggesting that Ca^2+^-induced expression of Cmk1 in fission yeast is the result of up-regulated transcription (19). In addition to the Ca^2+^-responsive elements, the *cmk1* promoter also contains the previously described calcineurin-dependent response element (CDRE) AGCCTC/GAGGCT motif (35). This motif is found at the 296–291 position upstream of ATG in the *cmk1* promoter (Figure 3A).

In the light of the above observations, we studied whether the increment in *cmk1* gene expression and protein levels is dependent on the Prz1 transcription factor in response to Ca^2+^. As shown in Figure 3B, the increase in *cmk1* mRNA in response to Ca^2+^ was abolished in *Dprz1* cells and the same was observed at the protein level (Figure 3C). Cmk1 was phosphorylated in *Dprz1* cells exposed to Ca^2+^, but the increase in protein levels was barely noticeable (Figure 3C).

### Cmk1 negatively regulates Prz1

**Figure 4.**
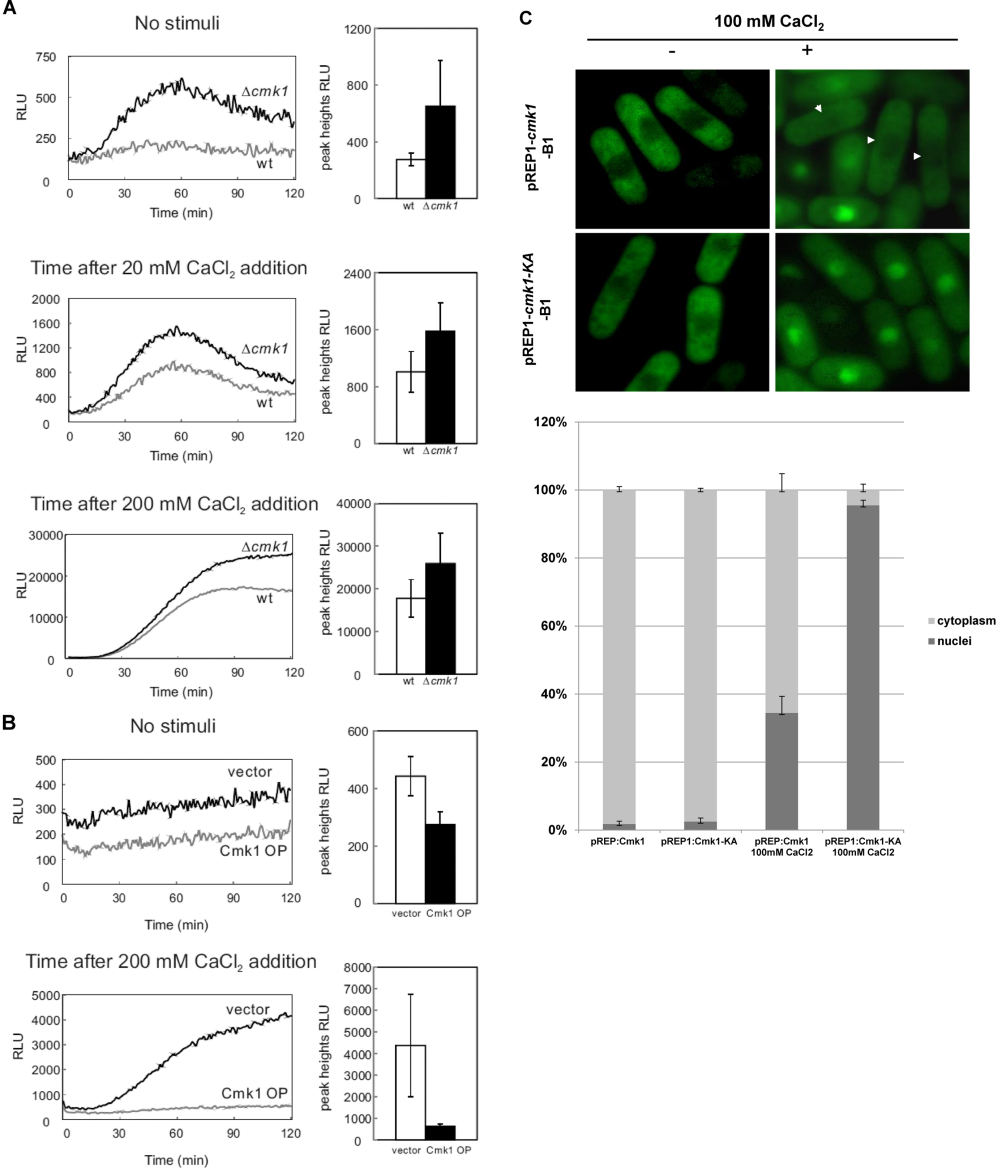
Cmk1 regulates Prz1 transcriptional activity. (**A**) Wild-type and *Δcmk1* strains transformed with the CDRE reporter vector were incubated with d-luciferun and untreated (no stimuli) and treated with either 20 mM or 200 mM CaCl_2_. Using a luminometer, light emission levels expressed as relative light units were measured up to 120 min. The graph ‘no stimuli’ represents the values of cells starting exponential growth. The data shown represent multiple experiments. (**B**) Wild-type cells harbouring the integration plasmid (CDRE-TATAbox-luc1) (KP2513 strain) were transformed with the multicopy plasmid empty (vector) or carrying the *cmk1* gene (Cmk1 OP). Cells were incubated with d-luciferin and treated with various concentrations of CaCl_2_ as indicated. As in (A) light emission levels expressed as relative light units were measured up to 120 min. The graph ‘no stimuli’ represents the values of cells starting exponential growth. The data shown represent multiple experiments. The graphics on the right in A and B represent the overall peak heights of the relative light units measured from the graphs on the left. (**C**) Cells expressing chromosomal *GFP-Prz1* under the control of the *nmt* promoter were treated (+) or untreated (-) with 100 mM CaCl_2_ and Cmk1 (pREP1-*cmk1*) or the inactive Cmk1-KA (pREP1-*cmk1-KA*) were overexpressed under the control of *nmt* promoter by growing the cells in EMM without thiamine (-B1). The graph represents the quantification of GFP-Prz1 localisation in each indicated condition of three different experiments.

In budding yeast and mammalian cells, the activity of Crz1 and NFAT transcription factors, is regulated by different signalling pathways which affect kinases (14). This prompted us to examine whether Cmk1 is involved in negative regulation of Prz1 by analysing the Ca^2+^-induced CDRE transcriptional activity of Prz1 in *Δcmk1* cells compared to wild-type. The reporter response of *Δcmk1* cells was markedly enhanced, reflecting the increase of Prz1 transcriptional activity compared with wild-type cells (Figure 4A). In contrast, overexpression of Cmk1 suppressed the Prz1 transcriptional activity (Figure 4B), indicating that Cmk1 is indeed involved in the negative regulation of Prz1.

In Ca^2+^-stimulated cells, active Prz1 accumulates in the nuclei; so we next studied whether the cellular location of Prz1 is affected by Cmk1. We overexpressed Cmk1 and Cmk1 kinase-dead (Cmk1-KA), which has the lysine 60 from the ATP-binding site mutated to alanine, in *GFP-prz1* cells in the presence of Ca^2+^. We observed that Prz1 nuclear localisation was altered, displaying a cytoplasmic pattern when Cmk1 was overexpressed, while Prz1 was maintained in the nuclei with Cmk1-KA expression (Figure 4C).

### Cmk1 interacts with and phosphorylates Prz1

**Figure 5.**
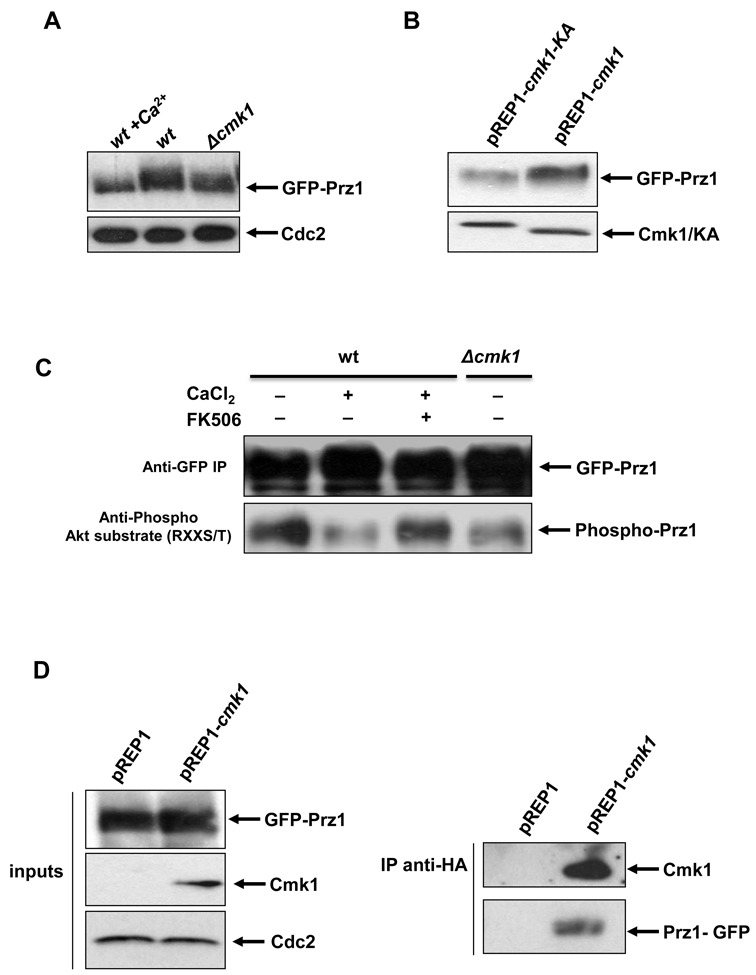
Cmk1 phosphorylates and binds Prz1. (**A**) Mobility shift of the Prz1 protein was analysed by Western blot using anti-GFP antibodies from *Δcmk1* cells *(Δcmk1*) and wild-type cells untreated (wt) or treated with 100 mM of CaCl_2_ (wt+Ca^2+^) carrying the endogenous GFP-prz1 tagged gene (top). Anti-PSTAIR antibody was used to probe Cdc2 as a loading control (bottom). (**B**) Cmk1 and Cmk1-KA were overexpressed in GFP-Prz1 cells harbouring pREP1-Cmk1 or pREP1-Cmk1-KA plasmids in the absence of thiamine. The Prz1 protein was analysed by Western blot using anti-GFP antibodies (top). The level of Cmk1 and Cmk1-KA overexpression was detected using the anti-HA antibodies (bottom). (**C**) *In vivo* Prz1 phosphorylation by Cmk1. Prz1 was immunoprecipitated using anti-GFP antibodies from *Δcmk1* and wild-type cells untreated (wt) or treated with 100 mM CaCl_2_ and 0.5 μg/ml of FK506 (top). Phosphorylation of Prz1 was detected with anti-Phospho Akt substrate (RXXS/T) (bottom). (**D**) Cmk1 protein was immunoprecipitated using anti-HA antibody from GFP-Prz1 cells harbouring the plasmid pREP1-Cmk1 or the empty plasmid pREP1 in the absence of thiamine (right top). Binding of Prz1 was detected using anti-GFP antibodies (right bottom). Left panels indicate the input levels of Prz1 (top), Cmk1 (middle) and Cdc2 as a loading control (bottom) detected with anti-GFP, anti-HA anti-PSTAIR antibodies, respectively.

Prz1 activation and subcellular localisation are regulated by its phosphorylation state. In response to Ca^2+^ stimulation, Prz1 is dephosphorylated and activated (18). This activation can be monitored by a change in electrophoretic mobility, in addition to nuclear accumulation. We analysed the Prz1 protein in *Δcmk1* cells and the mobility shift of Prz1 observed in wild-type cells is abolished in the absence of Cmk1 (Figure 5A). Moreover, we overexpressed Cmk1 and the inactive Cmk1-KA in wild-type cells. As Figure 5B shows, when Cmk1 was overexpressed, the Prz1 protein exhibited a slow electrophoretic-mobility which was absent with overexpression of Cmk1-KA, indicating that Prz1 is hyperphosphorylated when Cmk1 is overexpressed (Figure 5B).

We then analysed whether Cmk1 phosphorylates Prz1 *in vivo*. To detect Prz1 phosphorylation, we used an antibody against phospho-Akt substrates (RXXS/T) which recognises proteins containing phospho-serine/threonine preceded by arginine at the -3 position. This substrate specificity is characteristic of many Arg-directed kinases, including Cmk1 (36).

We then analysed the phosphorylation status of Prz1 by using the antibody against phospho-Akt substrates (RXXS/T). We immunoprecipitated endogenous GFP-Prz1 from wild-type cells treated or untreated with Ca^2+^ and we observed that Prz1 was phosphorylated in untreated wild-type cells and hypophosphorylated in cells treated with calcium, thereby verifying the specificity of the antibody toward the phosphorylation state of Prz1. In Ca^2+^-treated cells, dephosphorylation is due to calcineurin as was confirmed when cells treated with Ca^2+^ were incubated beforehand with FK506, a specific inhibitor of calcineurin (Figure 5C). When the Prz1 protein was analysed in *Δcmk1* cells, it was observed to present a hypophosphorylated state (Figure 5C). This result confirms that Cmk1 phosphorylates Prz1 and suggests that Cmk1 is involved in Prz1 inactivation.

We next examined the interaction between Cmk1 and Prz1 by co-immunoprecipitation. Cmk1 protein was immunoprecipitated from cells containing endogenous *GFP-prz1* and a plasmid overexpressing Cmk1 or an empty plasmid as a control. As Figure 5D shows, Prz1 was only present when associated with Cmk1.

### Absence of the *prz1* gene markedly suppresses the high Ca^2+^-resistance of *cmk1* deleted cells

**Figure 6.**
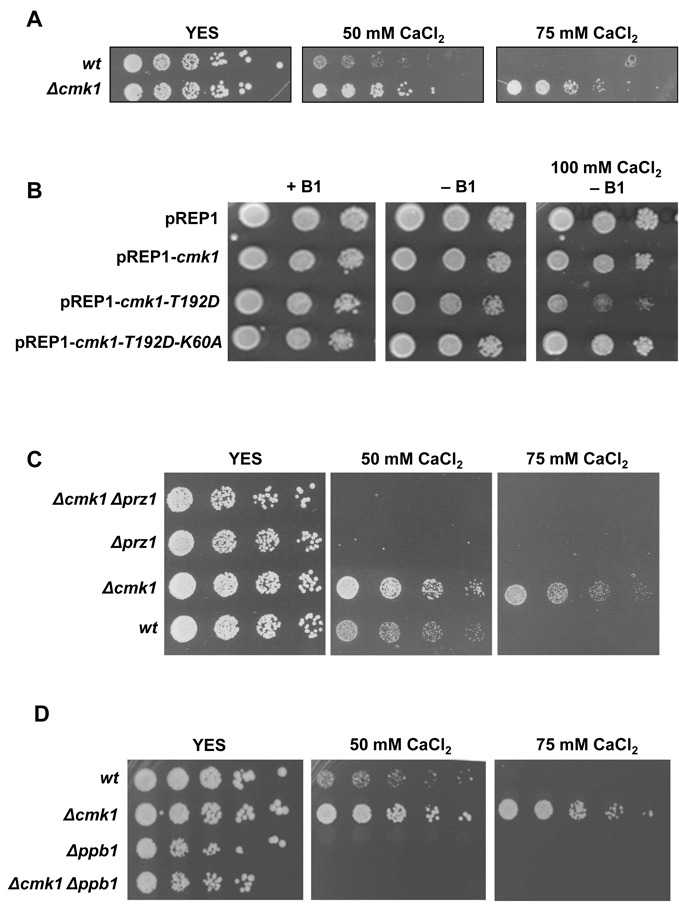
Suppression of Ca^2+^ resistance of *cmk1* deleted cells by elimination of calcineurin pathway. (**A**) Ca^2+^ sensitivity. Wild-type and *Δcmk1* cells were grown in YES medium and spotted on YES plates containing different concentrations of CaCl_2_ and incubated for 3 days at 30°C. (**B**) *Δcmk1* cells containing the multicopy plasmid pREP1, empty (pREP1) or carrying different versions of *cmk1* gene (pREP1-*cmk1*, pREP1-*cmk1-T192D* and pREP1-*cmk1-T192D-K60A*) were spotted onto EMM plates with thiamine (+B1), without thiamine (-B1) and without thiamine plus CaCl_2_ (-B1+ 100mM CaCl_2_) and were incubated for 4 day at 30°C. (**C**) and (**D**) The indicated strains were grown in YES medium and spotted on YES plates containing different concentrations of CaCl_2_ and incubated for 3 days at 30°C. Lower CaCl_2_ concentrations of figures C and D are shown in Supplementary Figure S1.

The above results imply a role of Cmk1 in the control of Ca^2+^ homeostasis. Therefore, we screened the growth of wild-type and *Δcmk1* cells at different concentrations of Ca^2+^. As shown in Figure 6A, *Δcmk1* cells displayed a resistant phenotype to high Ca^2+^ concentrations. This observation is consistent with its role as a negative regulator of Prz1 (Figure 6A). In contrast, overexpression of the constitutively active form of Cmk1 (Cmk1-T192D) was capable of suppressing Ca^2+^ resistance (Figure 6B). The sensitivity to Ca^2+^ acquired with Cmk1-T192D expression was associated with Cmk1 activity because overexpression of the catalytically inactive Cmk1-T192D, which has the lysine 60 from the ATP-binding domain mutated to alanine (Cmk1-T192D-K60A), rendered it insensitive to Ca^2+^ (Figure 6B).

To provide additional information about regulation between Cmk1 and Prz1, we analysed the sensitivity of double mutant *Δcmk1 Δprz1* cells at high doses of Ca^2+^. The Ca^2+^ resistance of *Δcmk1* cells was abolished in the double *Δcmk1 Δprz1* mutant (Figure 6C). These results suggest that the loss of Prz1 activity abolishes *Δcmk1* resistance to Ca^2+^; accordingly, the same should be observed in the absence of the Prz1 activator, calcineurin (*ppb1*). Therefore, we examined the double mutant *Δppb1 Δcmk1* at high Ca^2+^ concentrations and, as expected, deletion of *ppb1* abolished the *Δcmk1* Ca^2+^ resistance (Figure 6D). These results suggest that the activity of Prz1 is necessary to allow *Δcmk1* cells to grow in calcium conditions.

### Cmk1 induces cell-cycle arrest triggered by Cdc25 after Ca^2+^ treatment

**Figure 7.**
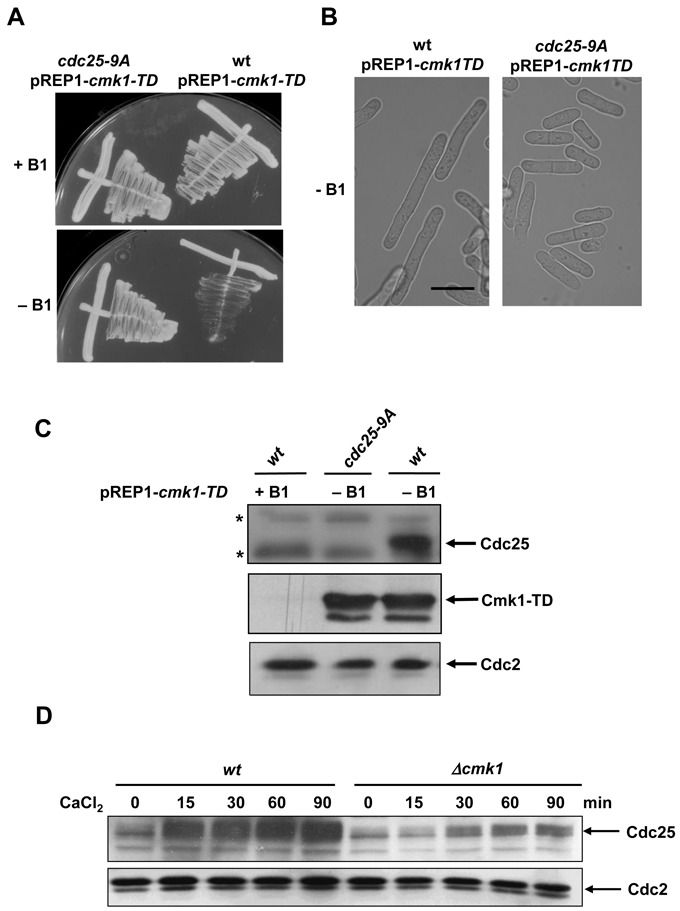
Overexpression of Cmk1 causes cell cycle arrest through Cdc25. (**A**) and (**B**) Wild-type (wt) and *cdc25–9A* cells containing the plasmid pREP1-*cmk1-TD* were grown either in the presence (+B1) or absence (-B1) of thiamine on (A) plates or (B) in liquid media. Bar, 10 μm. (**C**) Western blot of extracts from wild-type and c*dc25–9A* cells expressing pREP1-*cmk1-TD* grown either in the presence (+B1) or absence (-B1) of thiamine were probed with anti-Cdc25 antibodies (top), anti-HA antibodies to detect Cmk1-TD (middle) and anti-PSTAIR antibodies to detect Cdc2 as a loading control. * indicates unspecific bands. (**D**) Time course of wild-type (wt) and *Δcmk1* cells exposed to 100 mM CaCl_2_. The Cdc25 protein level was analysed at the times indicated by Western blot using anti-Cdc25 antibodies (top) and Cdc2 was probed as a loading control with anti-PSTAIR antibodies (bottom).

Expression of the constitutively active form of Cmk1 (Cmk1-T192D) arrests cell cycle progression (Figure 7A) (9). We and others have previously reported that activation of members of the CaM-dependent kinase family, such as Chk1, Cds1 or Srk1, causes cell cycle arrest dependent on Cdc25 phosphorylation (37–41). We therefore studied whether the cell cycle arrest caused by the overexpression of Cmk1-T192D is dependent on Cdc25 phosphorylation. To this end, we overexpressed Cmk1-T192D in a strain carrying the *cdc25–9A* allele, which contains mutations at 9 of the consensus sites of CaM-dependent kinases (40,42–43). The growth arrest and cell elongation typical of cell cycle arrest caused by the overexpression of Cmk1-T192D were suppressed when Cmk1-T192D was overexpressed in *cdc2–59A* cells (Figure 7A and B).

Next, we examined the Cdc25 protein when Cmk1 was expressed. As can be observed in Figure 7C, overexpression of the constitutively active Cmk1-T192D caused the stabilisation and accumulation of Cdc25. In contrast, the overexpression of Cmk1-T192D did not affect *cdc25–9A* cells. This result is consistent with our previous report (43), where Cdc25 was inhibited but stabilised through phosphorylation.

We then examined the Cdc25 protein level in wild-type and *Δcmk1* cells treated with Ca^2+^. As Figure 7D shows, Cdc25 protein levels accumulated during Ca^2+^ response in wild-type cells. In contrast, the Cdc25 protein failed to increase in *Δcmk1* cells during Ca^2+^ response (Figure 7D). Thus, Cdc25 accumulation caused by Ca^2+^ is dependent on Cmk1.

### The new CaMKK, Ckk2, phosphorylates Cmk1 in response to Ca^2+^ stress

Here, we addressed the critical question of how Cmk1 phosphorylation is regulated in response to Ca^2+^ stress. In mammalian cells, two-step activation of CaMK1 has been demonstrated; an increase in CaMK1 activity after Ca^2+^/CaM binding followed by hyperactivation through phosphorylation by CaMKK. In fission yeast, Cmk1 activity is Ca^2+^/CaM-dependent, and mutation of the putative phosphorylation site (Thr-192) for CaMKK to aspartic acid (Cmk1-T192D) converts Cmk1 into a constitutively active form (9), indicating that Cmk1 is regulated in a similar way to mammalian CaMKI.

Previous studies have postulated that Ssp1 is fission yeast CaMKK (12,44). Accordingly, we studied whether Ssp1 is involved in Cmk1 activation. However, Cmk1 was still phosphorylated in *ssp1*-deleted cells exposed to Ca^2+^ (Supplementary Figure S2A) and overexpression of Ssp1 did not display the phosphorylation pattern of Cmk1 (Supplementary Figure S2B). Regarding the Ca^2+^ stress response, *Δssp1* cells were highly sensitive to Ca^2+^, in contrast to the resistance of *Δcmk1* cells (Supplementary Figure S2C). Taken together, these results indicate that Ssp1 is not the kinase involved in Cmk1 regulation in the Ca^2+^ stress response.

**Figure 8.**
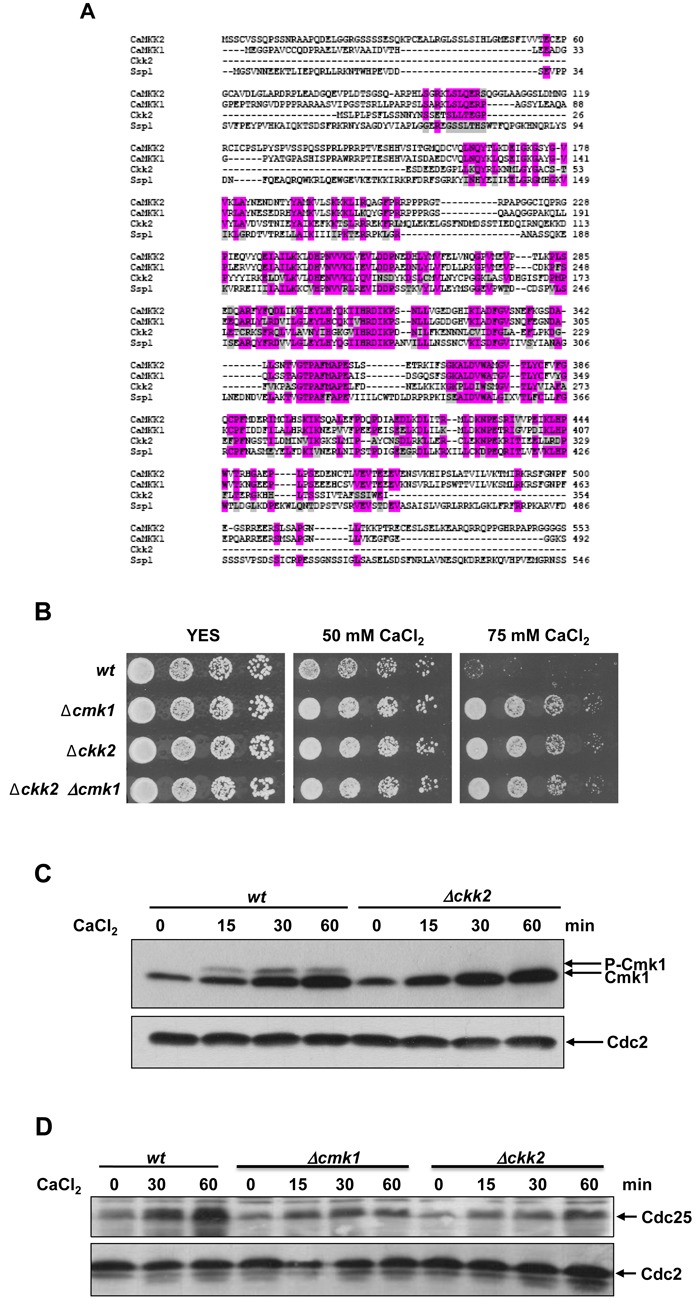
Ckk2 a new CaMKK in fission yeast. (**A**) Comparison of amino acid sequences of human and *S. pombe* CaMKK. Human CaMKK2 (CaMKK2), human CaMKK1 (CaMKK1), *S. pombe ckk2* (Ckk2) and *S. pombe ssp1* (Ssp1). Multiple sequences were aligned with Clustal Omega. (**B**) Ca^2+^ sensitivity. Wild-type (wt), *Δcmk1*, *Δckk2* and *Δcmk1 Δckk2* cells were grown in YES medium and spotted on YES plates containing different concentrations of CaCl_2_ and incubated for 3 days at 30°C. (**C**) Time course of wild-type (wt) and *Δckk2* cells exposed to 100 mM CaCl_2_. The Cmk1 protein and phosphorylation mobility shift were analysed at the times indicated by Western blot using anti-HA antibodies (top) and Cdc2 was probed as a loading control with anti-PSTAIR antibodies (bottom). (**D**) Time course of wild-type (wt), *Δcmk1* and *Δckk2* cells exposed to 100 mM CaCl_2_. The Cdc25 protein level was analysed at the times indicated by Western blot using anti-Cdc25 antibodies (top) and Cdc2 was probed as a loading control with anti-PSTAIR antibodies (bottom).

In addition to Ssp1, we found a second sequence homologous to mammalian CaMKK: SPC1919.01, named Ppk34 for the uncharacterised putative protein kinase 34 in a systematic deletion analysis of fission yeast kinases (45). Due to its homology to the CaMKK proteins, we have renamed it Ckk2. Ckk2 is most homologous to mammalian CaMKK2 (34%), followed by Ssp1 (32%) and CaMKK1 (28%) (Figure 8A and Table 1).

**Table 1.** Basic local alignment search tool for the Ckk2 (GenBank ID 162312133) protein with CaMKK, CaMKK2, CaMKK1 and Ssp1

Protein	Ckk2 cover	E value	Identities	GenBank ID
**CaMKK2**	86%	6e -52	34%	27437015
**Ssp1**	63%	2e -30	32%	19075860
**CaMKK1**	52%	4e -14	28%	14150045

Next, we examined the effect on the sensitivity to Ca^2+^ stress of single *ckk2* or double *ckk2 cmk1*-deleted cells and we observed that *Δckk2* and *Δcmk1 Δckk2* cells were resistant to Ca^2+^ stress as *Δcmk1* cells are (Figure 8B). This result suggests that Ckk2 is the CaMKK involved in Cmk1 regulation under high Ca^2+^ concentrations. Therefore, we examined Cmk1 phosphorylation in *Δckk2* cells under Ca^2+^ stress, and found that Cmk1 phosphorylation was lost in *Δckk2* cells (Figure 8C), indicating that the newly identified Ckk2 kinase is responsible for Cmk1 phosphorylation in response to Ca^2+^.

We also examined the Cdc25 protein level in *Δckk2* cells compared to wild-type and *Δcmk1* cells treated with Ca^2+^. Cdc25 protein levels in *Δckk2* cells were similar to those in *Δcmk1* cells and, failed to increase during Ca^2+^ response (Figure 8D), reinforcing the claim that Ckk2 is in the same pathway as Cmk1 during Ca^2+^ signalling.

## DISCUSSION

Yeast cells adapt to a wide range of growth conditions through activation of signalling pathways that detect and respond to changes in the environment. An increase in Ca^2+^ activates phosphatase calcineurin, promoting survival under these conditions in part by dephosphorylating the Prz1 transcription factor in fission yeast (18). Upon dephosphorylation, Prz1 translocates from the cytosol to the nucleus, where it activates gene expression. Activation of Prz1 must be reversible in order to maintain cell homeostasis. In budding yeast and mammalian cells, it has been shown that rephosphorylation of calcineurin-dependent transcription factors, Crz1 and NFAT, respectively, upon activation of gene expression is a key process in the Ca^2+^ response (46). In budding yeast, calcineurin dephosphorylates Crz1p and causes its rapid translocation from the cytosol to the nucleus. The casein kinase I isoform Hrr25, cyclic AMP-dependent protein kinase A (PKA) and Pho85 cycling-dependent kinase act to negatively regulate Crz1 (21,25,47). After adaptation to calcium stress, Crz1 relocalises from the nucleus to the cytoplasm. The phosphorylation status of Crz1 has been shown to influence this shuttling by altering its rates of nuclear import and export (48,49). Furthermore, the regulation of Crz1 by multiple kinases in budding yeast is analogous to the regulation of the mammalian functional homolog of Crz1, NFAT. Nuclear export of NFAT responds to phosphorylation by multiple kinases (46). Moreover, in human, cardiovascular diseases such as hypertrophy (50,51), neurodegenerative diseases such as Alzheimer's (51–53) and tumour progression by inducing angiogenesis (54–56), are promoted by persistent NFAT activation. Therefore, considerable research has focused on studying the mechanisms of negative regulation of calcineurin-dependent transcription factors.

In fission yeast, a genetic screen to isolate Prz1 regulators identified Pka1 kinase; however, cAMP had no effect on the electrophoretic mobility of Prz1, and phosphorylated Prz1 was not detected by phospho-PKA substrate-specific antibodies (30).

Our central goal was to elucidate the role of Cmk1 kinase in response to Ca^2+^. Here, we present evidence that Cmk1 is part of the feedback regulation whereby active Prz1 activates Cmk1 expression which in turns inactivates the Prz1 transcriptional function in response to an increase in cellular Ca^2+^. The expression of Cmk1 increases both at the mRNA and protein level in response to calcium, and Prz1 is the transcription factor involved in the *cmk1* mRNA increase. In addition, several lines of evidence indicate that Cmk1 is involved in the inactivation of Prz1 in response to Ca^2+^. First, the expression of Cmk1 prevents Prz1 nuclear localisation in response to Ca^2+^, thereby preventing the activation of Prz1-dependent transcription. Cmk1 activity is necessary to inhibit Prz1 because expression of the catalytically inactive form of Cmk1 (Cmk1-KA) does not affect Prz1 nuclear localisation. Second, Prz1 activity is higher in *cmk1* deleted cells and lower when Cmk1 is overexpressed. Third, Cmk1 binds and phosphorylates Prz1 *in vivo*. The overexpression of Cmk1 causes a change in the electrophoretic mobility of the Prz1 protein which is not observed when the inactive Cmk1-KA is overexpressed. Furthermore, phosphorylation in the consensus site of Cmk1 kinase is detected in the inactive Prz1, whereas this phosphorylation is absent in *Δcmk1* cells. Lastly and importantly, the cellular Ca^2+^-resistance of *Δcmk1* cells is suppressed by *prz1* deletion or by deletion of an upstream factor of Ca^2+^ signalling, such as calcineurin, *pbb1*, indicating that the lack of negative regulation of Prz1 and thus, the increase in transcription of its targets involved in the calcium response, confers Ca^2+^-resistance.

Prz1 activation tends to normalise intracellular Ca^2+^ levels through the expression of its targets. It was tempting to speculate that Cmk1 would be involved in controlling intracellular Ca^2+^ levels. Thus, we also studied whether Ca^2+^ uptake and cellular Ca^2+^ concentrations were affected by the absence of Cmk1. We have monitored a change in intracellular free Ca^2+^ concentrations with the Ca^2+^ sensor, yellow Cameleon-nano15 (57,58). Upon addition of Ca^2+^, images of CFP and YFP emission spectra were taken and the YFP/CFP ratio was calculated. The YFP/CFP ratio increased significantly in both wild-type and *Δcmk1* cells after Ca^2+^ treatment (Supplementary Figure S3), indicating that changes in intracellular Ca^2+^ levels are not sufficient to provide Ca^2+^-resistance despite the slight decrease in Ca^2+^ levels observed in *Δcmk1* cells.

In fission yeast, overexpression of Prz1 induced slow growth and small cell morphology similar to that of constitutively active calcineurin (30,59). We found that overexpression of Cmk1 counteracts the effect of Prz1 and the cells recover the wild-type phenotype (Supplementary Figure S4). This observation is interesting because previous studies were conducted on fission yeast to determine whether the overexpression of known kinases that oppose calcineurin-dependent activation, such as GSK3 homologues (GSk3 and Gsk31), DYRK family kinases (Pom1, Ppk15 and Prp4), p38 MAPK homologues (Sty1), PKC homologues (Pck2), ERK MAPK homologues (Pmk1) and pheromone responsive MAPK (Spk1), repress the phenotype caused by overexpression of Prz1 in fission yeast; but none of them did (30). However, despite the overexpression of Cmk1 rescues the slow growth phenotype induced by overexpression of Prz1, it is not sufficient to indicate that Cmk1 acts directly over Prz1, the effect could also be due to the cell cycle regulation by Cmk1.

In fission yeast, it has previously been reported that the constitutively active form of Cmk1 blocks cell cycle progression, although the mechanism remains elusive (9). We found that in response to an increase in calcium, Cmk1 blocks the cell cycle through phosphorylation of the Cdc25 protein phosphatase.

This result contributes to generalising a function already defined for members of the CaM-dependent kinase family, such as Chk1, Srk1 and MAPK-K2, which, in response to different genotoxic or environmental stresses, blocks cell division through phosphorylation of Cdc25 (37,41,60).

We found that Ca^2+^ signalling also provokes phosphorylation of Cmk1. As indicated, Cmk1 activity depends on Ca^2+^/CaM and full activation is reached upon phosphorylation at T192: the CaMKK consensus site (9). Therefore, the CaMKK Ssp1 was the candidate to phosphorylate Cmk1 in response to Ca^2+^. However, Ssp1 is not the kinase that phosphorylates Cmk1 in response to Ca^2+^. Cmk1 phosphorylation takes place in *Δssp1* cells exposed to Ca^2+^, and *Δssp1* cells are not resistant to high levels of Ca^2+^, consistent with having Cmk1 active. Therefore, we focused on a second gene of *S. pombe* showing homology to mammalian CaMKKs: SPCC1919.01, named Ppk34 as an uncharacterised putative protein kinase 34 in a systematic deletion analysis of fission yeast kinases (45). Due to its homology to the CaMKKs, we have renamed it Ckk2. We found that like *Δcmk1* cells, *Δckk2* and *Δcmk1 Δckk2* deleted cells are resistant to high concentrations of Ca^2+^, supporting the idea that Ckk2 shares the same pathway as Cmk1. An idea which is confirmed by our observation that Cmk1 phosphorylation is abolished in *Δckk2* cells exposed to Ca^2+^. Additionally, Ckk2 is also involved in Cdc25 stabilisation in response to Ca^2+^ stress corroborating the Ckk2-Cmk1 axis. Further experiments will be required to assess how Ckk2 is regulated.

In summary, we have identified a novel member of the CaM-dependent kinase family, the CaMKK, Ckk2, which is involved in the activation of Cmk1 by phosphorylation in response to Ca^2+^. Furthermore, we have also identified a novel physiological role for Cmk1 in regulating the calcineurin signalling pathway by phosphorylating Prz1.

**Figure 9.**
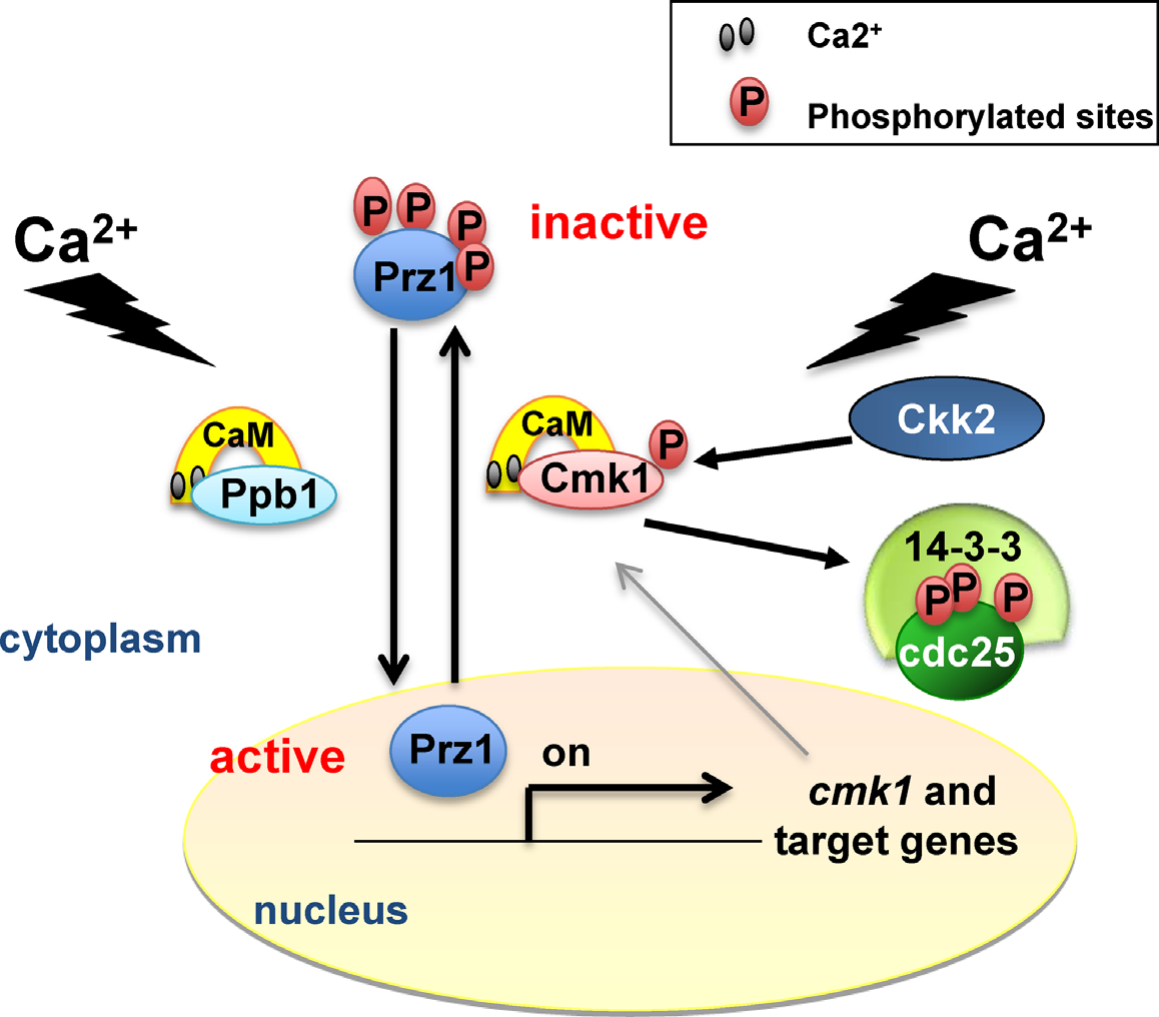
Model depicting the role of the Ckk2-Cmk1 pathway in Ca^2+^ response. An increase in intracellular Ca^2+^ levels results in activation of Ca^2+/^CaM-dependent proteins, including the phosphatase calcineurin (Ppb1) and the Ca^2+/^CaM-dependent kinases Cmk1 and Ckk2. Ppb1 dephosphorylates Prz1 leading to its nuclear translocation and activation of *cmk1* gene transcription. In parallel, activation of Ckk2 phosphorylates and fully activates Cmk1 which phosphorylates and inactivates both; Cdc25 and Prz1, promoting their nuclear export.

Under normal growing conditions, calcineurin (Ppb1), Cmk1 and probably Ckk2 are inactive (Figure 9), and under these circumstances, Prz1 remains in the cytoplasm, phosphorylated by Cmk1 and most probably by additional kinases, since the deletion of *cmk1* does not provoke the immediate translocation of Prz1 to the nucleus. In response to an increase in Ca^2+^, the Ppb1, Cmk1 and Ckk2 proteins are activated. In this scenario, active Ppb1 activates Prz1 by dephosphorylation and consequently Prz1 translocation to the nucleus to activate its target genes, one of which is *cmk1*. An increase in Ca^2+^ also activates Cmk1. Activation of Cmk1 kinase occurs in two steps, first by binding Ca^2+^/CaM and second through phosphorylation by Ckk2 (Figure 9). Active Cmk1 has two important functions during the Ca^2+^ response that maintain cell homeostasis: inhibition of cell cycle progression by phosphorylation of the Cdc25 protein (Figure 9) and inhibition of Prz1-dependent transcription, which can be toxic to the cell, through phosphorylation of Prz1 and subsequent exit from the nucleus (Figure 9). Thus, while both activation and inactivation of Prz1 is regulated by Ca^2+^, the kinetics is different. The activation by Ppb1 is faster than the following inactivation by Cmk1.

In mammalian cells the kinase CaMKII, a member of the CaM-dependent kinases, is also activated in response to Ca^2+^ inhibiting NFAT nuclear translocation by directly phosphorylating calcineurin. CaMKII mediated phosphorylation of calcineurin has been widely reported (61–63). Specifically, studies in cardiac myocytes have indicated that an increase of Ca^2+^ activates two cytoplasmic signals with opposite effects on calcineurin activity. The increased calcineurin-mediated NFAT nuclear translocation was the dominant effect of elevated Ca^2+^, with CaMKII acting as negative regulator. Together calcineurin and CaMKII determine NFAT nuclear translocation, hypertrophy and pro-survival signalling (62). Additionally, in the nervous system has been reported, that cytosolic Ca^2+^ signals mediate the bidirectional responses of growth cone, attraction or repulsion are induced by CaMKII and calcineurin, respectively. Interestingly, a relatively large local Ca^2+^ elevation preferentially activates CaMKII which induces attraction; while a modest local Ca^2+^ signal predominantly acts through calcineurin producing repulsion (63).

Our results also indicate that Ca^2+^ stress activates two cytosolic signalling cascades with opposite effects on Prz1 nuclear activity; the Pbb1 and the Ckk2/Cmk1 cascades. It would be interesting to study whether Cmk2 kinase is also involved in Pbb1 inhibition by direct phosphorylation and to determine whether there is a balance between Cmk2 and Pbb1 depending on the cytosolic Ca^2+^ level.

Moreover, due to the functional analogy, it should be interesting to study whether the CaMKK/CaMK1 axis regulates NFAT expression in addition to CaMKII in pathologies such as Alzheimer's disease, muscular hypertrophy or cancer progression and metastasis, where hyperactivation of NFAT has been detected (53,55,64–66).

## SUPPLEMENTARY DATA

Supplementary Data are available at NAR Online.

SUPPLEMENTARY DATA
