# Supplementary material for: Negative feedback regulation of calcineurin-dependent Prz1 transcription factor by the CaMKK-CaMK1 axis in fission yeast
Source: Nucleic Acids Res. 2014 Jul 31;42(15):9573–87. doi: 10.1093/nar/gku684 (PMC4150787; doi:10.1093/nar/gku684)
Supplement: SUPPLEMENTARY DATA [file supp_42_15_9573__index.html]

Negative feedback regulation of calcineurin-dependent Prz1 transcription factor by the CaMKK-CaMK1 axis in fission yeast — SUPPLEMENTARY DATA 

# Negative feedback regulation of calcineurin-dependent Prz1 transcription factor by the CaMKK-CaMK1 axis in fission yeast

## SUPPLEMENTARY DATA

**Files in this Data Supplement:**

- SUPPLEMENTARY DATA
