# Supplementary material for: Negative feedback regulation of calcineurin-dependent Prz1 transcription factor by the CaMKK-CaMK1 axis in fission yeast
Source: Nucleic Acids Res. 2014 Jul 31;42(15):9573–87. doi: 10.1093/nar/gku684 (PMC4150787; doi:10.1093/nar/gku684)

Supplementary Information

Supplementary Material and Metodhs

Measurement and Quantification of Cytosolic free Ca (2+) Levels Using Cameleon

Yeast cells expressing yellow Cameleon-nano 15 under the thiamine repressible promoter, *nmt1*, were grown at 30°C and treated with 100 mM $CaCl_2$ for 1 h 30 min. For live analysis, cells were immobilised on a glass slide using colourless lectins from *Glycine max* (Sigma, St Louis, MO). CFP and YFP emission images were viewed using a spectral confocal microscope (Leica TCS-SL). Image processing was performed using Image J software. The CFP:YFP ratio indicates cytosolic free Ca(2+) concentration. Data analysis was performed using the statistical software Prism 5 (GraphPad Software, Inc., San Diego, CA). The Student's t test was applied to compare data.

Supplementary Table S1. *Schizosaccharomyces pombe* strains

**Strain**	**Genotype**	**Source**
RA2501	*h−leu1-32 ura4-D18*	Lab stock
RA2542	*h−cmk1::hph leu1-32 ura4-D18*	This work
	h- cmk1::ura leu1-32 ura4-D18	*Bimbo et al. 2005*
RA2663	*h− cmk1-HA:kanMX6 leu1-32 ura4-D18*	This work
RA2705	*h− prz1::kanMX6 leu1-32 ura4-D18*	Lab stock
RA2726	*h− cmk1-HA:kanMX6 prz1::kanMX6 leu1-32 ura4-D18*	This work
RA2736	*h− cmk1::hph prz1::kanMX6 leu1-32 ura4-D18*	This work
RA2833	*h− p3-nmt-GFP-prz1:kanMX6 leu1-32 ura4-D18 pREP1-cmk1-K60A*	*This work*
	h- ckk2::ura4+ leu1-32 ura4-D18	*Bimbo et al. 2005*
RA3001	*h- ckk2::ura4+ cmk1-HA:kanMX6 leu1-32 ura4-D18*	This work
RA3001	*h- ppk34::ura4+ cmk1-HA:kanMX6 leu1-32 ura4-D18*	This work
MA6	*h- ssp1-XN::ura4+ leu1-32 ura4-D18*	*Matsusaka et al. 1995*
RA2725	*h- ssp1-XN::ura4+ cmk1-HA:KanMX6 leu1-32 ura4-D18*	This work
RA1652	*h- ssp1-XN::ura4+ cmk1::kan leu1-32 ura4-D18*	This work
RA2805	*h−leu1-32 ura4-D18 pREP1-YC2*	This work
RA2807	*h−cmk1::hph leu1-32 ura4-D18 pREP1-YC2*	This work
RA3042	*h−cmk1::hph leu1-32 ura4-D18 pREP1*	This work
RA2809	*h−cmk1::hph leu1-32 ura4-D18 pREP1-cmk1*	This work
RA1753	*h−cmk1::hph leu1-32 ura4-D18 pREP1-cmk1-T192D*	This work
RA1755	*h−cmk1::hph leu1-32 ura4-D18 pREP1-cmk1-T192D-K60A*	This work
SP1938	*h- ppb1::kan leu1-32*	*Dr. R. Sugiura*
RA2944	*h- cmk1::ura ppb1::hph leu1-32 ura4-D18*	This work
RA3031	*cdc25-9A:ura pREP1-cmk1-T192D*	This work
RA2729	*h− cmk1-HA:kanMX6 leu1-32 ura4-D18 ssp1-XN::ura4+ pREP1-ssp1*	This work
RA2731	*h− cmk1-HA:kanMX6 leu1-32 ura4-D18 ssp1-XN::ura4+ pREP1-ssp1KA*	This work
KP2513	*h−* CDRE-TATAbox-luc1::ura4+ leu1-32 ura4-D18	*Deng et al. 2006*

Supplementary Table S2. Oligonucleotides used in this work

**Oligonucleotides used for strain construction**	
Cmk1 pFA6a Fw	5'-GCACCATCTAACAATACAATAATTTACATTAAGAGAAACCTTTTACGTTTACCTTCGTTGGCATTGGCGGATCCCCGGGTTAATTAA-3'
Cmk1 pFA6a Rv	5'-CGTCAACAGTCTAACAATGTCAGAAAACTGGTAGCGTTACTTTTTAAAACATCGACATAACTTTTAGCATTTTGTATGAGGC GAATTCGAGCTCGTTTAAAC-3'
Cmk1 pFA6a -3HA Fw	5'-CGTTCCGTACTGCCTATAACGCTGTACGTGCTTTCAACACTTGGAAAAAGCTTGAGAATAAGCAT CGGATCCCCGGGTTAATTAA-3'
Prz1 pFA6a Nmt GFP Fw	5'-GATACTTTTGGTAATTAATTATTCATTCCACTAATAAATTTCACACCCTTGGTGTTGCTGGTTTATCCAATTTATTACTAGAATTCGAGCTCGTTTAAAC-3'
Prz1 pFA6a Nmt GFP Rv	5'-TTTGAAAGGTTATCGTATAAAGAAGAAGGATTCAAGTCTTTGAATCTACGATTCGCTTCTTCTGACCTTTGACGCTCCATTTTGTATAGTTCATCCATGC-3'
**Oligonucleotides used for plasmid construction**	
Cmk1 XhoI Fwd	5'-CGCGCGCCTCGAGGTATGCAGCAAACATACAAACCAAACACCTCGGCC-3'
Cmk1 NotI BamHI Rv	5'-CGCTGTACGTGCTTTCAACACTTGGAAAAAGCTTGAGAATAAGCATTGCGGCCGCTAAGGATCCCGCGCG-3'
Cmk1 K60A Fwd	5'-CGAAACTAACAAAATGTATGCGGCTGCGATTATGAATAAAAAGATGATGG-3'
Cmk1 K60A Rv	5'-CCATCATCTTTTTATTCATAATCGCAGCCGCATACATTTTGTTAGTTTCG-3'
Cmk1T192D Fwd	5'- CTCAATACTATATGCTCATGGACGCATGTGGGACACCAGAG-3'
Cmk1T192D Rv	5- CTCTGGTGTCCCACATGCGTCCATGAGCATATAGTATTGAG-3
**Oligonucleotides used for mRNA monitorization by Real-Time**	
Cmk1 RT Fw	5'-GAGTACATGGCACCCGAAG-3'
Cmk1 RT Rv	5'-TGAGAAGGTCGAGCAAATGG-3'
Prz1 RT Fw	5'-GAGGATACTCTTGCGTCTGAAG-3'
Prz1 RT Rv	5'-CATTCCCGGTTCGAGGATAAG-3'
Act1 RT Fw	5'-CGCCGAACGTGAAATTGTTCGTGA-3'
Act1 RT Rv	5'-TCAAGGGAGGAAGATTGAGCAGCA-3'

Supplementary Figure S1. Suppression of Ca^{2+} resistance of *cmk1* deleted cells by elimination of calcineurin pathway. **(A)** Wild-type, *Δcmk1, Δprz1 and* the double *Δprz1Δcmk1* cells were grown in YES medium and spotted on YES plates containing different concentrations of $CaCl_2$. **(B)** Wild-type, *Δcmk1, Δppb1 and* the double *Δppb1Δcmk1* cells were grown in YES medium and spotted on YES plates containing different concentrations of $CaCl_2$. Plates were incubated for 3 days at 30°C.

Supplementary Figure S2. Cmk1 regulation is independent of Ssp1 kinase.

(A) Time course of wild type (wt) and *Δssp1* cells exposed to 100 mM $CaCl_2$. The Cmk1 protein and phosphorylation mobility shift was analysed at the times indicated by Western blot using anti-HA antibodies (top) and Cdc2 was probed as a loading control with anti-PSTAIR antibodies (bottom).

(B) Ssp1 and Ssp1 catalytically inactive (Ssp1-KA) were overexpressed in *cmk1-HA* cells harboring pREP1-Ssp1 or pREP1-Ssp1-KA plasmids in absence of thiamine. Cmk1 protein was analysed by Western blot using anti-HA antibodies (middle). The level of Ssp1 and Ssp1-KA overexpression was detected using the anti-HA antibodies (top) and Cdc2 was probed as a loading control with anti-PSTAIR antibodies (bottom).

(C) Ca^{2+} sensitivity. Wild type (wt), *Δcmk1*, *Δssp1* and *Δcmk1 Δspp1* cells were grown in YES medium and spotted on YES plates containing 50mM $CaCl_2$ and incubated for 3 days at 30°C.

Supplementary Figure S3. Intracellular free Ca^{2+} was measured using yellow Cameleon-nano 15 and quantifying the YFP/CFP ration in wild type and *Δcmk1* cells untreated (wt and *Δcmk1*) or treated with 100 mM $CaCl_2$ (wt+Ca^{2+} and *Δcmk1* +Ca^{2+}).

Supplementary Figure S4. Cells overexpressing GFP-Prz1 from the gene chromosomally integrated under the *nmt* promoter (*nmt-GFP-prz1*) were transformed with the multicopy plasmid expressing Cmk1 under the control of *nmt* promoter (pREP1-*cmk1-HA*) and were grown on EMM without thiamine (-B1) to induce the *nmt* promoter for 24 h at 30°C. Cells were examined under the microscope. Bar 10 μM.

Figure S1

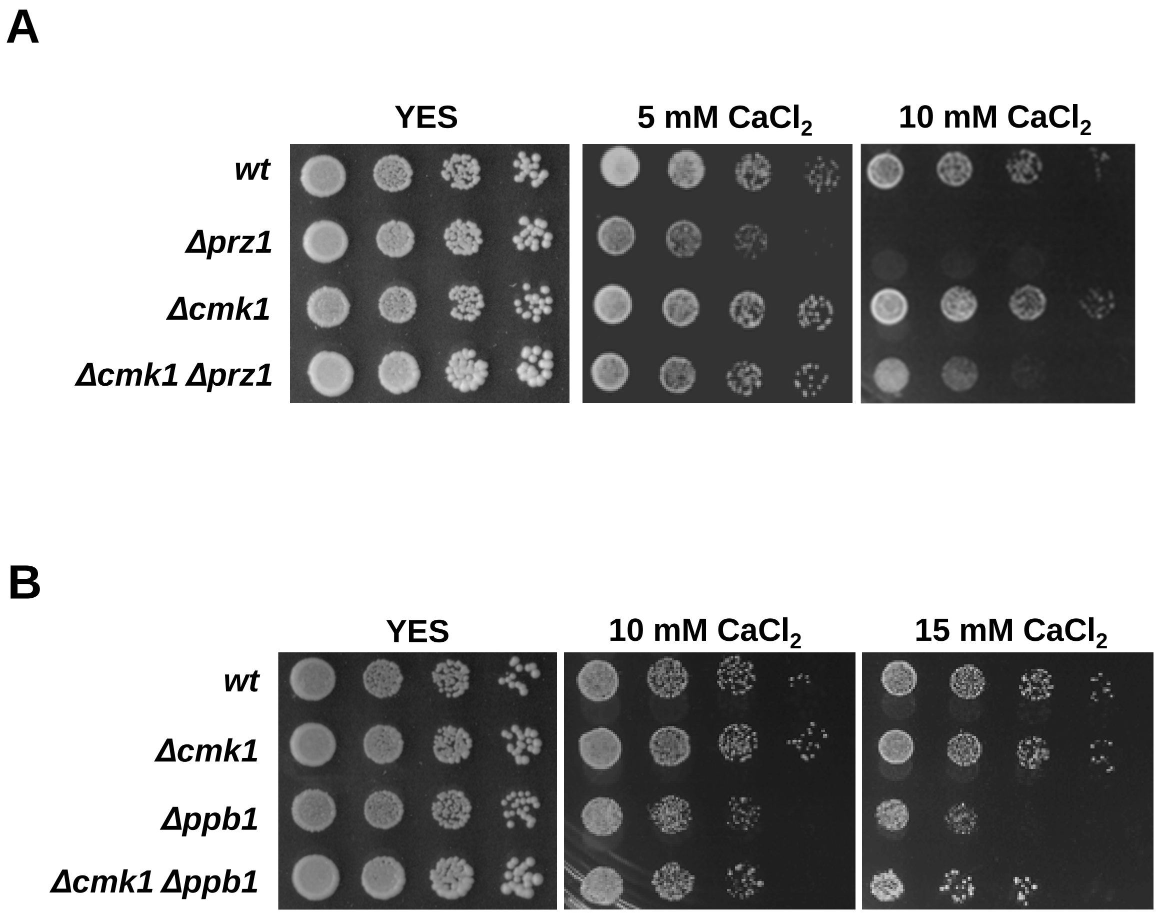
A
YES
5 mM CaCl2
10 mM CaCl2
wt
Δprz1
Δcmk1
Δcmk1 Δprz1
B
YES
10 mM CaCl2
15 mM CaCl2
wt
Δcmk1
Δppb1
Δcmk1 Δppb1

Figure S2

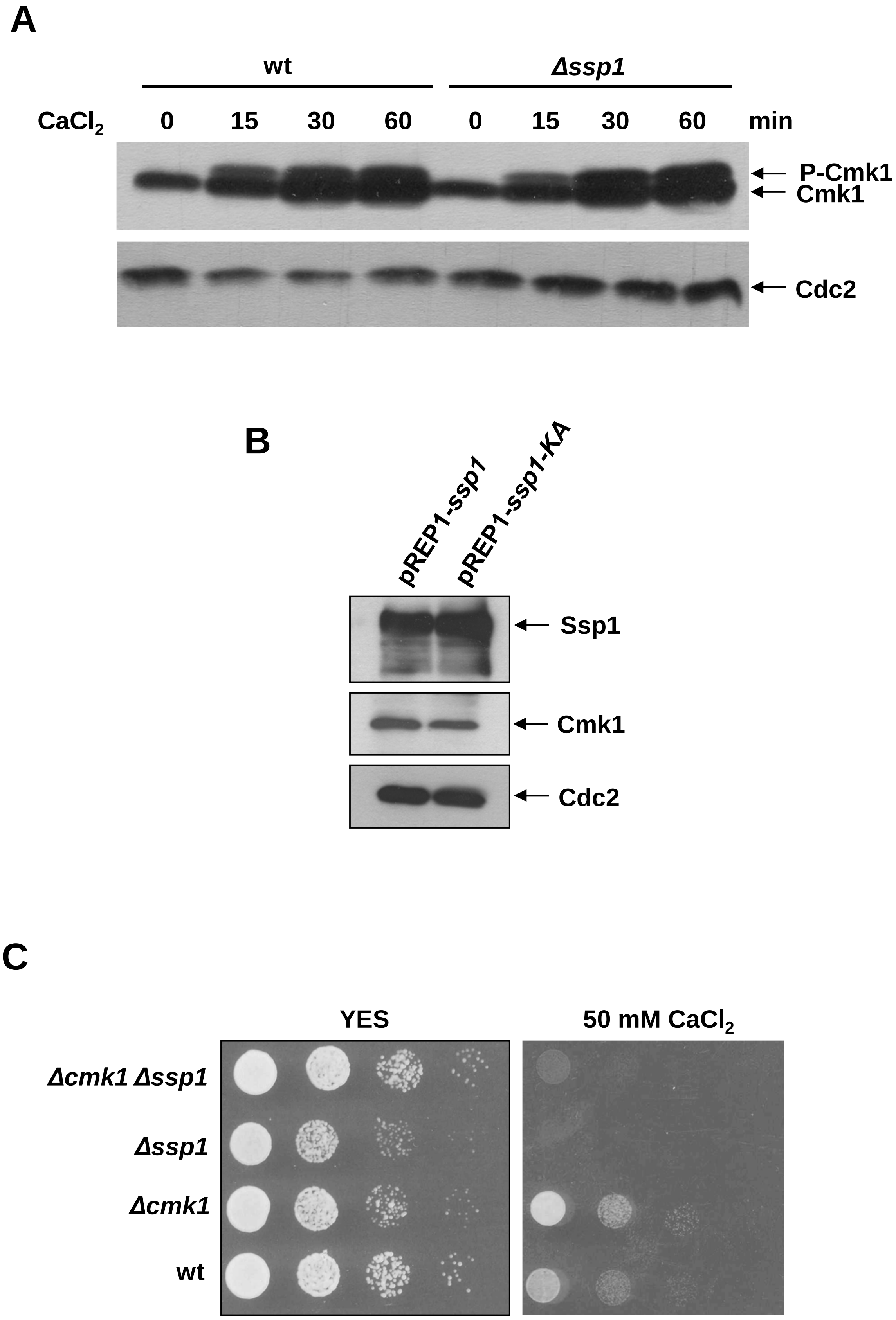
A
wt
Δssp1
CaCl2
0
15
30
60
0
15
30
60
min
P-Cmk1
Cmk1
Cdc2
B
pREP1-ssp1
pREP1-ssp1-KA
Ssp1
Cmk1
Cdc2
C
YES
50 mM CaCl2
Δcmk1 Δssp1
Δssp1
Δcmk1
wt

Figure S3

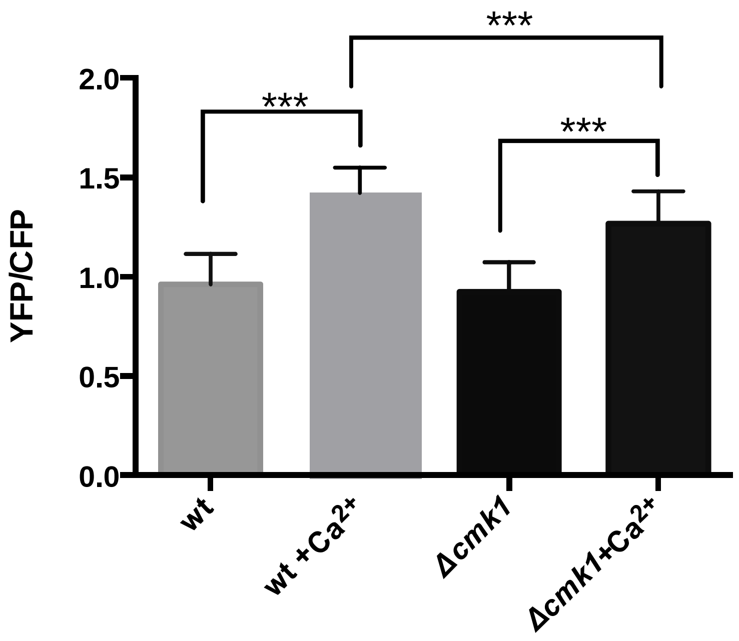

Figure S4

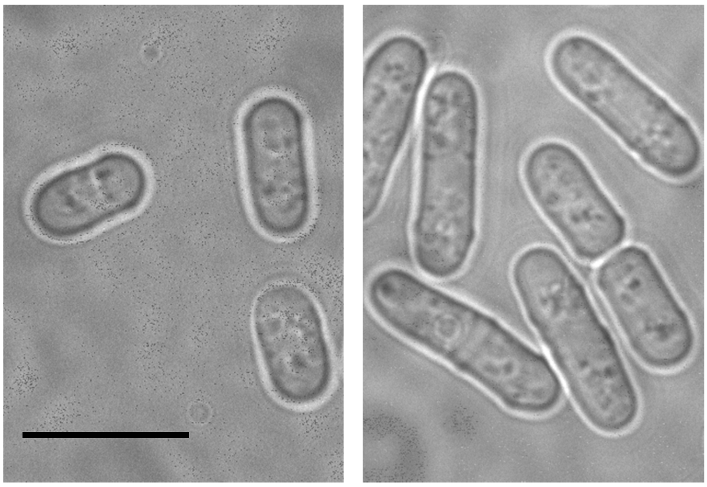

Supplement: SUPPLEMENTARY DATA [file supp_gku684_nar-01375-x-2014-File011.pdf]
